# Production of oligomeric procyanidins by mild steam explosion treatment of grape seeds

**DOI:** 10.1186/s40643-021-00376-4

**Published:** 2021-03-23

**Authors:** Jie Zhang, Dan Liu, Aoke Wang, Li Cheng, Wenya Wang, Yanhui Liu, Sadeeq Ullah, Qipeng Yuan

**Affiliations:** 1 College of Life Science and Technology, Beijing University of Chemical Technology, Beijing, 100029 China; 2 Amoy-BUCT Industrial Biotechnovation Institute, Beijing University of Chemical Technology, Amoy, 361022 China

**Keywords:** Polymeric procyanidins, Oligomeric procyanidins, Steam explosion, Depolymerization, Antioxidant activities

## Abstract

**Background:**

Sixty five percent of procyanidins in grape seeds is polymeric procyanidins (PPC), and they could not be assimilated directly by human. To enhance procyanidin assimilation, steam explosion treatment (SE) was used to facilitate the preparation of oligomeric procyanidins (OPC) from grape seeds.

**Results:**

The results indicate that SE treatment made grape seeds loose and porous, and decreased the mean degree of polymerization (mDP) of procyanidins. The procyanidins content and total phenolic content (TPC) were decreased with the increase of SE severity, while the amount of catechin (CA), epicatechin (EC) and epicatechin-3-O-gallate (ECG) were increased, resulting in significant increase of antioxidant activity.

**Conclusions:**

Although SE treatment could depolymerize PPC and produce CA/EC/ECG with high yield, it caused the yield loss of total procyanidins. SE treatment is a potential effective method to prepare procyanidins with low degree of polymerization and high antioxidant activity. However, it still needs to study further how to balance the yield of total procyanidins and catechin monomers (CA/EC/ECG).

**Supplementary Information:**

The online version contains supplementary material available at 10.1186/s40643-021-00376-4.

## Introduction

Grape pomace is the major solid residues from wine and grape juice industries, which mainly consist of grape seeds and skins (Silvan et al. 2020). Proanthocyanidins are distributing widely in skins and seeds of grape (Souquet et al. 1996; Prieur et al. 1994), and proanthocyanidins in grape seeds were procyanidin-type, consisting of catechin (CA) and epicatechin (EC) units, with partial galloylation (Porter 1988; Spranger et al. 2008). Procyanidins of grape seeds were constructed with C4-C8 linkages being much more abundant than C4-C6 bonds during the polymerization of flavanol units (Monagas et al. 2010; Unusan 2020), and the degree of polymerization may be reached around 30 (Hayasaka et al. 2003; Spranger et al. 2008). Procyanidins show a wide biological activity, such as antivirus, radical scavenging, anti-oxidation and anti-tumor (Hümmer and Schreier 2010). Sixty five percent of procyanidins in grape seeds are polymeric procyanidins (PPC) with degree of polymerization (DP) > 4 (Luo et al. 2018) and they could not be assimilated directly by human due to the higher degree of polymerization (Ou and Gu 2014). Therefore, if PPC could be depolymerized into oligomeric procyanidins (OPC), bioavailability and bioactivity of procyanidins could be increased significantly (Meeran and Katiyar 2007)

The available methods for depolymerizing PPC can be classified as: (1) acid hydrolysis with nucleophiles, such as phloroglucinol (Matthews et al. 1997) and benzyl mercaptane (Gu et al. 2002), while the depolymerizing reagents were not allowed to apply in food, pharmaceutical and cosmetic industry due to the toxicity of nucleophiles; (2) acid hydrolysis with expensive chain breakers, such as catechin (CA), epicatechin (EC), and epicatechin-3-O-gallate (ECG) (Liu et al. 2013); (3) acid hydrolysis with sulphurous acid, which produced the procyanidins with higher yield and purity, but its potential environmental impact is a big problem (Luo et al. 2018); (4) acid salt hydrolysis, such as sodium bisulfite, which also confronts the serious environmental problem (Lin et al. 2014); (5) alkaline hydrolysis, which only converted 6% of the PPC into OPC (White et al. 2010); (6) hydrogenolysis, which needs dangerous operating conditions and requires expensive catalysts (Li et al. 2015). Taken together, the earlier reported methods indicated some drawbacks for their industrial applications, such as toxic byproducts, low hydrolyzing rate, dangerous operating conditions, high cost-consumption and environmental pollution (Wu et al. 2019). As a result, it is essential to develop an economic and environmental-friendly process for the production of OPC from PPC.

Steam explosion (SE) is an inexpensive and clean thermophysico-chemical process, including physical smash and chemical hydrolyzation (Jacquet et al. 2015). During steam explosion, the raw material was broken into pieces, and the micropores in original plant tissue were destroyed intensively (Chen and Chen 2011). Many natural products, such as flavonoids (Chen and Chen 2011; Qin and Chen 2015), total phenolic compounds (Gong et al. 2012; Serrano et al. 2017; Conde et al. 2009) and keratin (Zhang et al. 2015b), have been extracted with the aid of SE treatment. SE was used to increase the yield of flavonoids (quercitrin) by 8 times, and quercitrin was deglycosylated by cleavage of C-O bonds under SE treatment (Chen and Chen 2011). SE treatment could influence plant tissue in two ways: physical effect (destroying the structure of plant tissue) and chemical effect (chemical reaction at higher temperature including hydrolyzation, oxidation, pyrolysis, etc.) (Carvalheiro et al. 2008; Alvira et al. 2010). The physical effect could make the plant tissue loose and porous (larger cavities and intercellular spaces) by destroying the matrix of plant tissue, which could enhance the extracting rate of natural products (Carvalheiro et al. 2008; Alvira et al. 2010). The chemical effect could produce the natural products or release the natural products from their conjugates by the cleavage of the chemical bonds (Carvalheiro et al. 2008; Alvira et al. 2010). After the plant tissue was treated by SE, the resultant extracts depend on operating conditions: in the case of hemicellulose, mild SE conditions favour recovery of the longer chain structures, while more severe conditions facilitate the formation of monomers and their degradation products (Wojtasz-Mucha et al. 2017). Since natural products were usually sensitive to higher temperature and longer time at higher temperature, mild SE was suitable for plant tissues pretreatment during preparation of natural products.

In the present paper, mild SE treatment was used to facilitate the extraction of oligomeric procyanidins from grape seeds. The extracting conditions and antioxidant activity of procyanidins were investigated. The optimum SE operating conditions were determined. Finally, the depolymerization mechanism of procyanidin under SE treatment was suggested.

## Materials and methods

### Chemicals and raw material

CA and Folin − Ciocalteu phenol reagent were purchased from Sigma-Aldrich Chemical Co, Ltd, (St. Louis, MO, USA). EC, ECG, procyanidin B1, procyanidin B2 were purchased from Chengdu Manset Biotechnology Co, Ltd, (Chengdu, Sichuan, China). Procyanidin B3 and procyanidin C1 were purchased from Shanghai yuanye Bio-Technology Co, Ltd, (Shanghai, China). Cinnamtannin A2 was purchased from Shanghai ZZBIO Co, Ltd, (Shanghai, China). Gallic acid (GA), 2–20-azino-di-(3-ethylbenzthiazoline sulfonic acid) (ABTS) were purchased from TCI (Shanghai) Development Co, Ltd, (Shanghai, China). Vanillin, benzyl mercaptane, 2,2-diphenyl-1-picrylhydrazyl hydrate (DPPH) and 6-hydroxy-2,5,7,8-tetramethylchroman-2-carboxylic acid (Trolox) were purchased from Shanghai Macklin Biochemical Co, Ltd, (Shanghai, China). T-AOC Assay Kit was provided by Beyotime Biotechnology Co, Ltd, (Shanghai, China). Unless stated otherwise, the solvents used for chromatography were of high-performance liquid chromatography grade (HPLC-grade) and the other chemicals were of analytical reagent grade (AR-grade). Water was purified using a Milli-Q water purification system (Milford, MA, USA).

Grape seeds of “Chardonnay” were purchased from Xi'an Haoxuan Biotechnology Co, Ltd. The grape seeds were washed with deionized water for 3 times, then they were ground into powder by a high-speed blender and freeze-dried at − 40 °C in a vacuum freeze drier (SCIENTZ-18 N, Sunway Hanguang Electric Manufacturing Limited, Ningbo, China). The freeze-dried powder of grape seeds was sieved with a 20-mesh sieve, then sealed with N_2_ and kept in -20 ºC for further experiments.

### Steam explosion pretreatment, structural observation and procyanidins extraction

SE was carried out according to Zhang et al (2014). 100 g of freeze-dried grape seeds powder was mixed with 200 mL deionized water and the mixture was kept for 60 min at room temperature. The mixture was put into the SE equipment and pretreated at the combination of different pressures (0.3, 0.6, 0.9, 1.2 or 1.5 MPa) and different time-courses (30 s or 60 s), and then the pressure was reduced abruptly to atmospheric pressure. After SE, the pretreated grape seeds were collected and freeze-dried.

The unexploded and exploded samples were treated by dehydration, drying and coating with gold. Then, these samples were analyzed by scanning electron microscopy (SEM), (Japan Electronics Co, Ltd, Japan), which was operated at an accelerating voltage of 15 kV.

The extracting process of procyanidins was modified according to previous reports (Gu et al. 2002; Hellström and Mattila 2008). Two grams of freeze-dried unexploded and exploded samples were weighed. Then, they were defatted for 12 h with 20 mL hexane in 50 mL centrifuge tubes. After centrifugation at 9000 rpm for 10 min, the supernatants were discarded. The centrifuge tubes were placed into fume hood for 12 h to remove residual hexane. One gram of defatted samples was mixed with 10 mL of acetone/water/acetic acid (70/29.5/0.5, v/v/v) mixture in a 50 mL centrifuge tubes. The tubes were vortexed for 30 s, and then kept at 37 ℃ for 10 min with ultrasound treatment. Procyanidins were extracted for 50 min at ambient temperature under 300 rpm shaking. After centrifugation at 9000 rpm for 15 min, 7.5 mL supernatants were pipetted out and filtered using a polypropylene filter membrane (0.45 µm) for further analysis.

### Procyanidins content assessment

Procyanidins content assay was modified according to previous reports (Çam and Hışıl 2010), using an ultraviolet–visible V-5100B spectrophotometer (Shanghai Metash Instruments Co, Ltd, China). One milliliter of 60-fold diluted extract was first mixed with 2.5 mL of 1% vanillin in methanol solution, and then mixed with 2.5 mL of 25% H_2_SO_4_ in methanol solution. After keeping in water bath at 30 ℃ for 15 min, the absorbance of mixtures was read at 500 nm. Pure methanol was used as a blank. CA was used as a standard control, and the calibration curve has a good linear relationship at the range: 10–100 µg/mL *(R*^*2*^ = 0.9998). According to a calibration curve, the results were described as catechin equivalents (CE) (µg CA /g dry matter (DM)).

### Total phenolic content (TPC) assessment

TPC was determined according to the early-reported method (Xu et al. 2014) using an ultraviolet–visible V-5100B spectrophotometer (Shanghai Metash Instruments Co, Ltd, China). One hundred microliters of 40-fold diluted extracts were placed into 50 mL centrifuge tubes. Subsequently, 3900 µL of distilled water, 250 µL of 2 mol/L Folin–Ciocalteau reagent and 750 µL of 20% Na_2_CO_3_ were added. The mixtures were kept in water bath for 15 min at 30 ℃. Then, the absorbance of mixtures was read at 760 nm. Pure methanol was used as a blank control, gallic acid was used as a standard control and the calibration curve has a good linear relationship at the range: 100–800 µg/mL (*R*^2^ = 0.9999). According to a calibration curve, the final results were described as gallic acid equivalents (GAE) (µg GA/g DM).

### Mean degree of polymerization (mDP) assessment

The thiolysis of procyanidins was modified according to previous paper (Gu et al. 2002). Fifty microliters of extracts were mixed first with 50 µL 3.3% HCl in methanol solution in a 250-µL polypropylene insert, and then 100 µL 5% benzylmercaptan in methanol were added. The 250-µL polypropylene insert was placed into a 1.5-mL vial and the vial was quickly sealed with a cap. The mixtures were kept in water bath at 40 ℃ for 30 min, and then reacted at room temperature for 10 h for complete degradation. The final mixtures were kept at − 20 ℃ and measured by reversed phase high performance liquid chromatography (RP-HPLC). Thiolysis products of procyanidin B1, procyanidin B3, and the CA, CA benzylthioether, the EC, EC benzylthioether were used as standards to identify their counterparts (Furuuchi et al. 2011). Standard solutions for CA, EC, ECG, procyanidin dimers B1 and procyanidin dimers B3 were prepared at 1000 µg/mL.

Chromatographic analysis was modified according to previous papers (Gu et al. 2002). HPLC analysis was performed using a Shimadzu LC-20AT (Shimadzu, Japan) equipped with a UV-detector, a binary pump, an auto-sampler and a column compartment. Separation was carried out using a Diamonsil C18 column (5 μm, 250 × 4.6 mm i.d.) from Dikma Technologies (Beijing, China). The detection wavelength and column temperature were set at 280 nm and 35 ℃. The mobile phases were the mixture of solvent A (water/acetic acid = 98/2) and solvent B (methanol, 100%), which was developed as 0 min (A = 85%, B = 15%) → 45 min (A = 20%, B = 80%) → 50 min (A = 20%, B = 80%) → 55 min (A = 85%, B = 15%) → 70 min (A = 85%, B = 15%). The flow rate was set at 1.0 mL/min and the injection volume was set as 20 μL. Then, standard solutions were used to identify and quantify the samples. Although the presence of EGC-thiol and ECG-thiol was ignored in the early reports for mDP calculation (Furuuchi et al. 2011), their peaks were included in the present paper to increase the accuracy of the mDP calculation. The mDP of extracts can be calculated with the Eq. (1):

1 $$\mathrm{mDP}=\frac{Total\, area\, ratio\, of\, extension\, units\, benzlthioether}{Total\, area\, ratio\, of\, terminal\, units}+1$$

### Measurements of antioxidant activity

DPPH• radical-scavenging capacity, ferric reducing power and ABTS•^+^ radical-scavenging capacity were used to analyze the antioxidant activity of extracts. Diluted extract was prepared at same concentration (µg CA/g DM).

The DPPH assay was modified according to the reported papers (Xu et al. 2014). One hundred microliters of diluted extracts were placed into the tube. Then, 3900 µL of 25 µg/mL DPPH in methanol solution was added. The mixtures were kept in water bath for 60 min at 30 ℃ in darkness. Finally, the absorbance of mixtures was read at 515 nm. The scavenging capacity of diluted extracts on DPPH can be calculated with the following equation:

2 $$\mathrm{Scavenging\,rate}=\frac{({A}_{0}-\mathrm{A})}{{A}_{0}}\times 100 (\%)$$

where *A*_0_ was the absorbance of blank control (methanol solution); A was the absorbance of the diluted extracts. Trolox was used as a standard control, and the calibration curve has a good linear relationship between scavenging rate and concentration of Trolox solutions at the range: 100–1000 µmol/L (*R*^2^ = 0.9996). According to a calibration curve, the final radical scavenging activities were described as Trolox equivalent (TE) (µmol T/g DM).

The FRAP assay was carried out with a T-AOC Assay Kit (Xia et al. 2017). The experiments of the FRAP assay was performed in 96-well polystyrene microplates. Pure methanol solution was used as a blank control. Trolox was used as a standard control, and the calibration curve indicated a significant linear relationship between ferric reducing power and concentration of Trolox solutions at the range: 150–1500 µmol/L (*R*^2^ = 0.9983). According to a calibration curve, the final ferric reducing power was described as TE (µmol T/g DM).

The ABTS assay was carried out according to early reports (Oldoni et al. 2016). The 7 mmol/L ABTS and 140 mmol/L potassium persulfate were reacted for forming ABTS•^+^ radical at 25 ℃ in darkness for 12 h. To get absorbance value of 0.70 at 734 nm, the prepared ABTS•^+^ radical solution was diluted with ethanol. Thirty microliters of diluted extracts were mixed with 3.0 mL of the diluted ABTS•^+^ radical solution and kept at 30 ℃ in darkness for 6 min. At the end, absorbance of mixture was measured at 734 nm. Pure ethanol was as a blank. The radical scavenging capacity of the diluted extracts on ABTS•^+^ can be calculated with the following equation:

3 $$\mathrm{Scavenging\, rate}=\frac{({A}_{0}-\mathrm{A})}{{A}_{0}}\times 100 (\%)$$

where A_0_ was the absorbance of blank; A was the absorbance of diluted extracts. Trolox was used as a standard control, and the calibration curve has a good linear relationship between scavenging rate and concentration of Trolox solutions at the range: 100–2000 µmol/L (*R*^2^ = 0.9959). According to calibration curve, the final radical scavenging activities was described as TE (µmol T/g DM).

### NP-HPLC analysis of procyanidins

Chromatographic analysis was modified according to the early paper (Choy et al. 2013). Normal phase high performance liquid chromatography (NP-HPLC) analysis was carried out by a Shimadzu LC-20AT (Shimadzu, Japan). Separation was carried out on a Develosildiol 100 column (5 μm, 250 × 4.6 mm i.d.) from Nomura Chemical Co, Ltd, (Japan). The mobile phase was the mixture of solvent A (acetonitrile/water = 98/2, v/v) and solvent B (methanol/water/acetic acid = 95/3/2, v/v/v). The gradient elution program was developed as: 0 min (A = 93%, B = 7%) → 3 min (A = 93%, B = 7%) → 53 min (A = 62.4%, B = 37.6%) → 56 min (A = 0%, B = 100%) → 69 min (A = 0%, B = 100%) → 75 min (A = 93%, B = 7%) → 85 min (A = 93%, B = 7%). The flow rate was 1.0 mL/min and the injection volume was 20 μL. The detection wavelength and column temperature were set at 280 nm and 30 ℃. The peaks of procyanidins with different DP were assigned by comparing retention time with the standards (Additional file 1: Table S1) (Choy et al. 2013). Standard solutions of CA, EC, ECG, procyanidin B1, procyanidin B2, procyanidin B3, procyanidin C1 and Cinnamtannin A2 were prepared with concentration of 1000 µg/mL. The solutions of the standards were filtered using a polypropylene filter membrane (0.45 µm). One milliliter of 1 mg/mL CA, EC, ECG standards were mixed in screwed glass tubes to get procyanidin standards 1. One milliliter of 1 mg/mL procyanidin B1, procyanidin B2, procyanidin B3 standards were mixed to get procyanidin standards 2. One milliliter of 1 mg/mL CA, EC, ECG, procyanidin B1, procyanidin B2, procyanidin B3, procyanidin C1 and innamtannin A2 standards were mixed to get procyanidin standards 3. Standard solutions were used to quantify chemicals. Peak area growth ratio of procyanidins (RAGR) with different DP can calculated with the following equation:

4 $$\mathrm{RAGR}=\frac{{A}_{s-n}}{{A}_{u-n}}\left(\mathrm\,{n}=1,\mathrm{ECG}, 2, 3, 4, \ge 5\right)$$

where A_s-n_ was peak area of steam-exploded procyanidins with DP = n; A_u–n_ was peak area of unexploded procyanidins with DP = n.

### Statistical analysis

Statistical analysis was carried out with Origin 2017. Means were calculated using one sample *t*-Test. Significance level was set at P ≤ 0.05.

## Results and discussion

### Effects of SE treatment on grape seed structure and procyanidins content

**Fig. 1:**
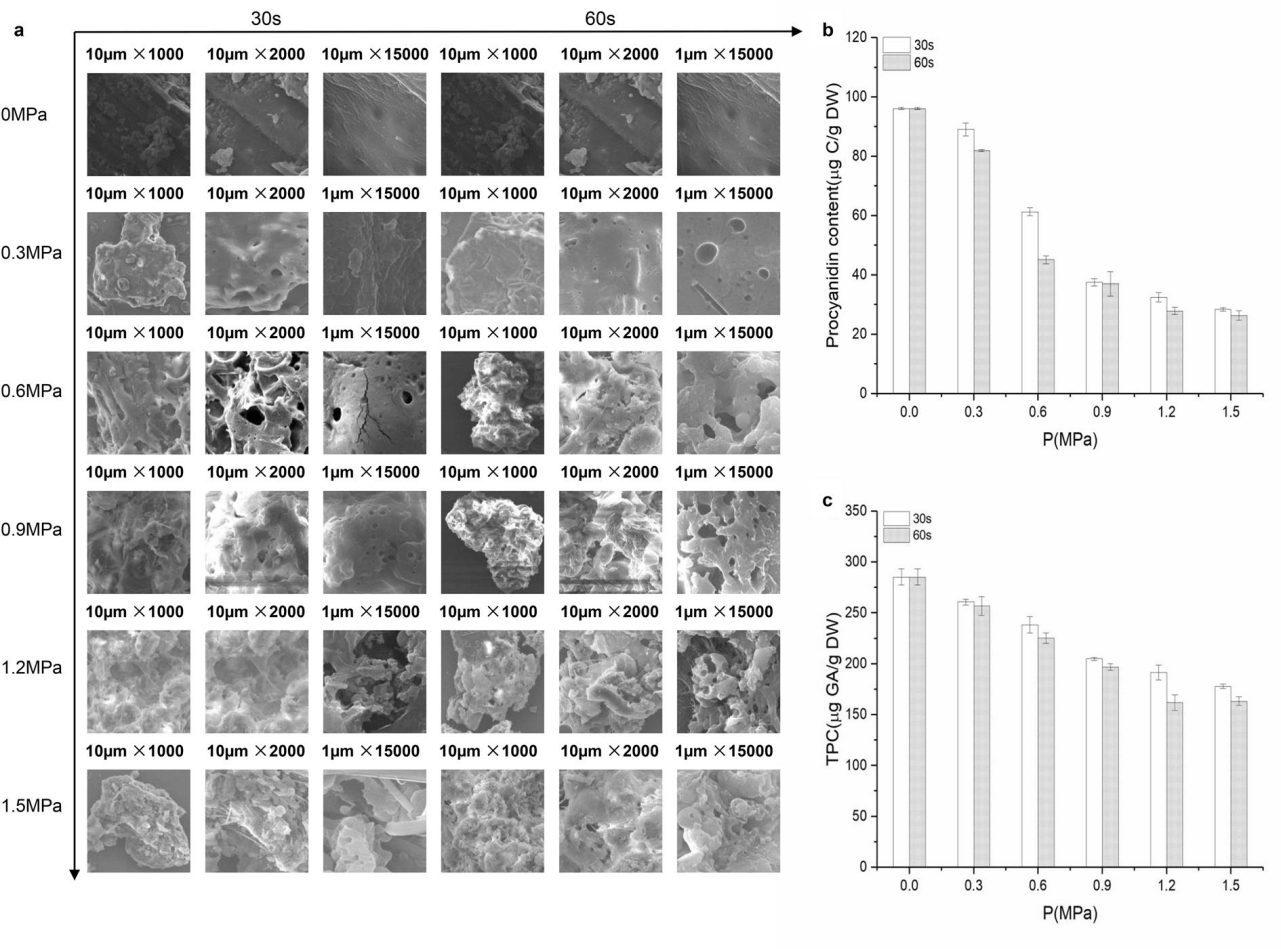
Structural and chemical changes after SE treatment. SEM images of the unexploded and exploded samples via SE treatment with different times and pressures (**a**). Effect of SE treatment on the procyanidins content (**b**) and total phenolic content (**c**)

The SEM micrographs of exploded and unexploded grape seeds are shown in Fig. 1a. From top to bottom, the SE pressures were varied from 0 to 1.5 MPa. From left to right, the operating time was kept for 30 s or 60 s. The SEM micrographs indicated the difference between the unexploded and exploded grape seeds. After the SE treatment, grape seeds became loose and porous, resulting in the larger cavities and more intercellular spaces (Wang et al. 2016).

However, Fig. 1b indicates that SE treatment did not increase the yield of procyanidins, but decreased the yield. It has been reported that the increase of SE severity decreased flavonoids yield due to procyanidins degradation (Qin and Chen 2015). When the pressure was increased from 0 to 1.5 MPa for 30 s or 60 s, the procyanidins content decreased from 96.12 to 28.37 µg CA/g DM or 96.12 to 26.35 µg CA /g DM, respectively. To evaluate whether the procyanidins were converted into other phenolic compounds or not, the TPC was measured and results are shown in Fig. 1c. The results indicated that TPC decreased significantly with the increase of severity, which did not agree with the reported results from barley bran (Gong et al. 2012). When the pressure increased from 0 to 1.5 MPa for 30 s or 60 s, the TPC decreased from 285.34 to 177.73 µg CA /g DM or 285.34 to 161.91 µg CA /g DM, respectively. The possible reason might be attributed to structural difference of plant tissues and phenolic compounds between barley bran and grape seeds, and the phenolic compounds in grape seeds might be more sensitive to SE treatment. Although the results couldn’t explain the reason of procyanidins loss, they indeed proved that the SE treatment could affect the yield of procyanidins. Therefore, the effect of SE treatment on procyanidins polymerization was investigated further.

### Effect of SE treatment on mean degree of polymerization (mDP) and antioxidant activity

**Fig. 2:**
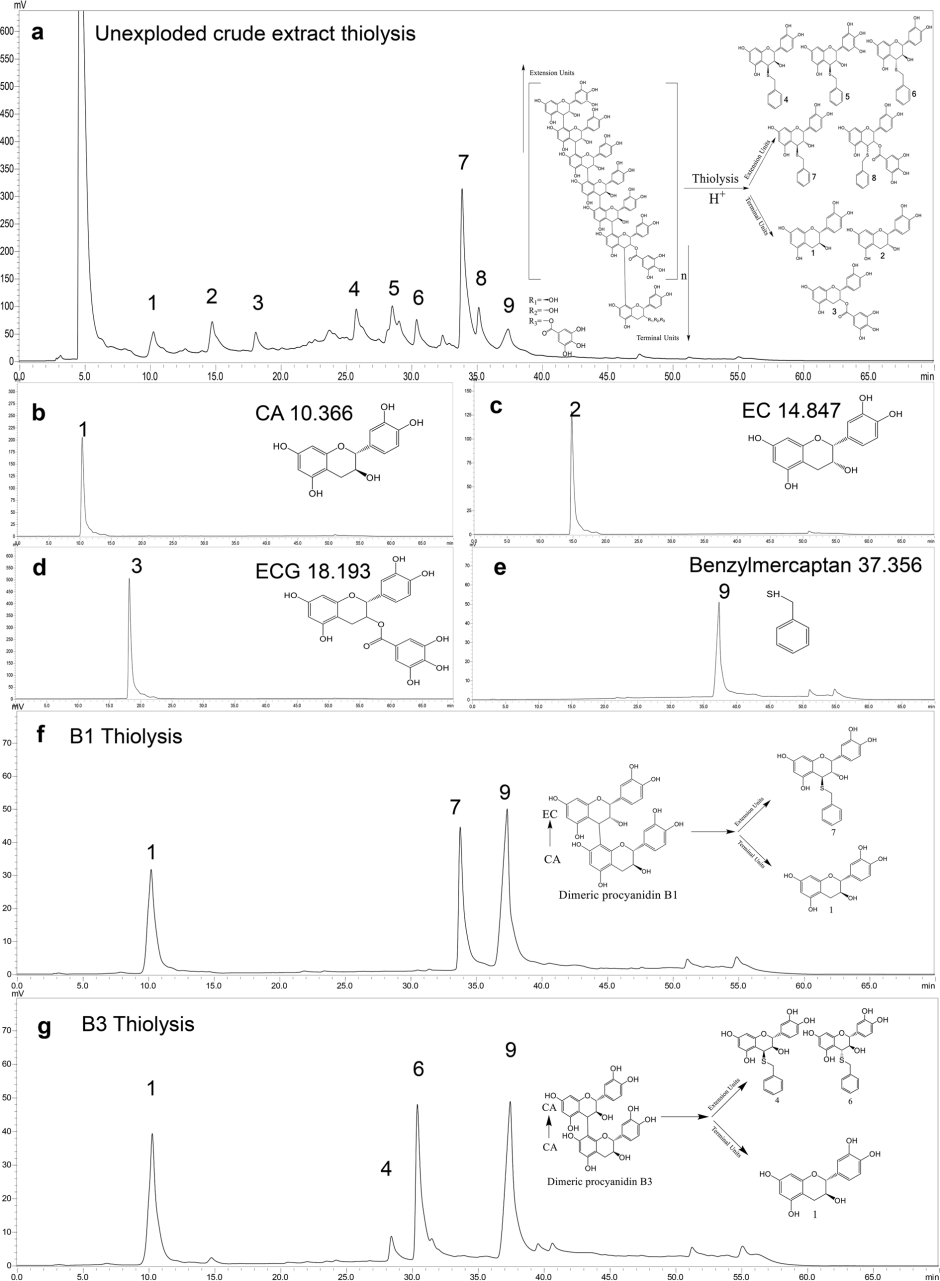
RP-HPLC chromatogram of thiolysed crude extract of unexploded grape seeds (**a**); terminal units: **b** CA (1), **c** EC (2), **d** ECG (3); **e** benzylmercaptan (9); extension units: **f** EC-thiol (7); **g** β-CA-thiol (4), α-CA-thiol (6)

Thiolysis was used to analyze the structure of procyanidins in different SE treatment conditions. In thiolysis reactions, the extension units of procyanidins were attacked by benzyl mercaptan to form benzylthioether, while the terminal units were released as a free procyanidin unit without the linkage of benzylthioether (Fig. 2a) (Gu et al. 2002). Among the thiolysis residues of procyanidins of grape seeds, 3,4-*trans*-catechin benzylthioether (β-CA-thiol), 3,4-*cis*-catechin benzylthioether (α-CA-thiol), 3,4-*trans*-epicatechin benzylthioether (EC-thiol), (epi) gallocatechin benzylthioether (EGC-thiol) and epicatechin-3-O-gallate benzylthioether (ECG-thiol) were derived from extension units, and CA, EC and ECG belonged to terminal units (Fig. 2), which agreed with earlier reports (Bordiga et al. 2011; Vivas et al. 2004). They were all identified, assigned and quantified with reference material and documents (Ito et al. 2013; Hellström and Mattila 2008) (Fig. 2, Additional file 1: Table S2).

Table 1 indicates that terminal unites of procyanidins from grape-seeds’ extract consisted of CA, EC and ECG, and the extension unites included CA, ECG, EC, EGC. The mDP was ranging from 5.03 to 1.97 with the increase of pressure and treating time, which indicated the negative relationship between mDP and SE severity. On the basis of the above results, it was indicated that the SE treatment could effectively depolymerize PPC into OPC.

**Table 1:** mDP of grape seeds procyanidins after SE treatments

P	T	Terminal units (%)				Extension units (%)				mDP
		CA	EC	ECG	β-CA	EGC	EC	ECG	α-CA	
0	–	2.15 ± 0.02a	2.70 ± 0.01a	1.26 ± 0.00a	5.10 ± 0.01a	3.69 ± 0.01a	10.21 ± 0.01a	2.67 ± 0.01a	2.89 ± 0.01a	5.03 ± 0.00a
	–	2.15 ± 0.02a	2.70 ± 0.01a	1.26 ± 0.00a	5.10 ± 0.01a	3.69 ± 0.01a	10.21 ± 0.01a	2.67 ± 0.01a	2.89 ± 0.01a	5.03 ± 0.00a
0.3	30	3.31 ± 0.01c	3.31 ± 0.01c	2.61 ± 0.01c	1.50 ± 0.01c	0.64 ± 0.01c	19.27 ± 0.01b	9.50 ± 0.01b	3.37 ± 0.01c	4.72 ± 0.01b
	60	3.38 ± 0.01c	3.50 ± 0.01b	2.81 ± 0.01c	1.48 ± 0.01c	0.61 ± 0.01c	20.29 ± 0.02c	9.45 ± 0.00b	3.10 ± 0.01c	4.61 ± 0.00b
0.6	30	2.99 ± 0.01c	2.45 ± 0.01c	1.82 ± 0.01c	3.86 ± 0.02b	–	10.83 ± 0.01b	5.12 ± 0.01b	5.40 ± 0.00b	4.47 ± 0.01b
	60	3.35 ± 0.01c	2.33 ± 0.00c	1.69 ± 0.01c	2.16 ± 0.01c	–	7.71 ± 0.01c	3.03 ± 0.01c	4.60 ± 0.01b	3.37 ± 0.00c
0.9	30	1.97 ± 0.01c	1.60 ± 0.00c	1.05 ± 0.01c	0.80 ± 0.01c	0.27 ± 0.02c	5.40 ± 0.00b	1.92 ± 0.02c	2.13 ± 0.02c	3.27 ± 0.01c
	60	2.59 ± 0.01c	1.69 ± 0.01c	1.05 ± 0.01c	0.97 ± 0.01c	–	3.16 ± 0.01c	1.05 ± 0.01c	2.59 ± 0.01c	2.46 ± 0.01c
1.2	30	2.05 ± 0.01b	1.27 ± 0.01c	0.72 ± 0.00c	0.63 ± 0.01c	–	1.33 ± 0.02c	0.31 ± 0.01c	1.64 ± 0.02c	1.97 ± 0.01c
	60	1.63 ± 0.01c	1.13 ± 0.01c	0.61 ± 0.00c	0.62 ± 0.01c	–	1.35 ± 0.01c	0.59 ± 0.01c	1.13 ± 0.02c	2.10 ± 0.00c
1.5	30	1.69 ± 0.01c	1.25 ± 0.01c	0.63 ± 0.01c	0.35 ± 0.01c	–	2.28 ± 0.01c	0.79 ± 0.02c	0.93 ± 0.02c	2.22 ± 0.03c
	60	1.58 ± 0.01c	1.21 ± 0.01c	0.65 ± 0.00c	0.38 ± 0.01c	–	2.62 ± 0.01c	0.90 ± 0.01c	1.02 ± 0.01c	2.43 ± 0.01c

*P* Pressure (MPa), *T* Time of steam-explosion treatment (s). The results are described as the means ± standard deviation (*n* = 3). Means in the same column with different letters indicate significant differences, which were determined by One sample *t*-Test. (c:significance at *P* < 0.01; b:significance at *P* < 0.05; a:significance at *P* > 0.05.)

**Fig. 3:**
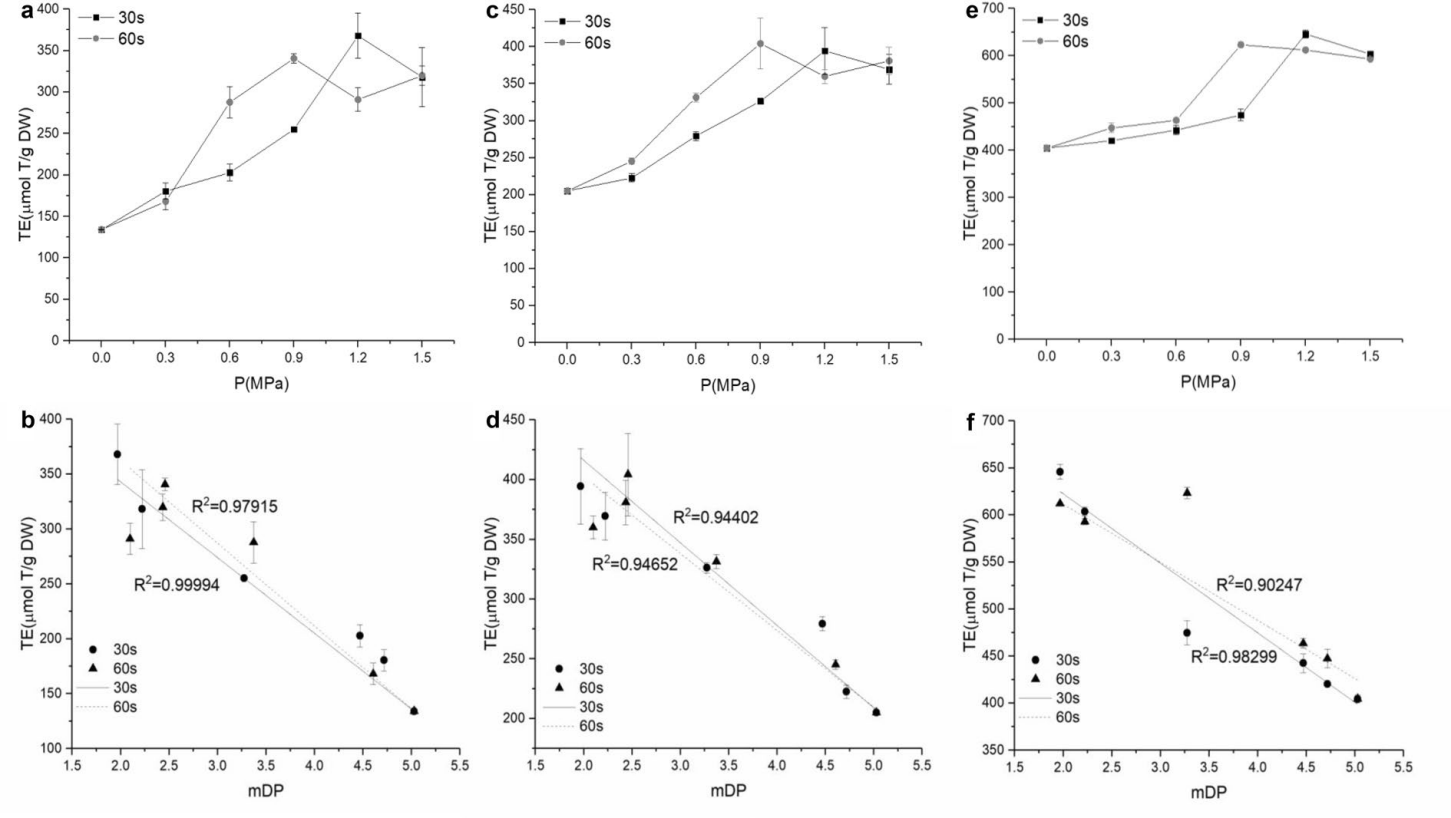
Antioxidant activity of the extracts with different SE treatments using the method of **a** DPPH, **c** FRAP and **e** ABTS. The correlation coefficients between mDP and **b** DPPH assay, **d** FRAP assay and **f** ABTS assay

It has been reported that OPC showed higher antioxidant activity in vitro or in vivo (Sun et al. 2011; Zhang et al. 2015a). The antioxidant activities of the procyanidins were found to be in a decreasing order: procyanidin C1 > procyanidin B2-3′-O-gallate > procyanidin B2 > procyanidin B1 > ECG > EC > CA > Trolox (Li et al. 2016; Luo et al. 2016). Furthermore, the antioxidant activities of procyanidins increased with the decrease of DP. Thus, the antioxidant activities of the different samples were analyzed by DPPH, ABTS and FRAP assay, which were described with TE (Fig. 3a, c, e). The antioxidant activities of SE-treated samples all increased significantly in comparison with the unexploded one. However, there was no positive relationship between the SE severity and antioxidant capacity, the higher correlations were found between antioxidant activity and mDP (Fig. 3b, d, f), with the correlation coefficient varying between 0.90247 and 0.99994 (*P* < 0.05). Moreover, the antioxidant activity was highly positively correlated with mDP value. The maximum antioxidant activity of DPPH, FRAP and ABTS assays were obtained at 1.2 MPa/30 s (368.00, 394.33, 645.96 µmol T/g DM) and 0.9 MPa/60 s (340.65, 404.22, 623.24 µmol T/g DM).

### Identification of procyanidin components after SE treatment by NP-HPLC

Chromatographic analysis was performed on the basis of the reported method (Choy et al. 2013) with the exception of replacing the fluorescence detectors with UV detectors. Although fluorescent detector was very sensitive for procyanidins analysis, the relative response factors of procyanidins with UV detector showed a better result (Chen et al. 2009).

**Fig. 4:**
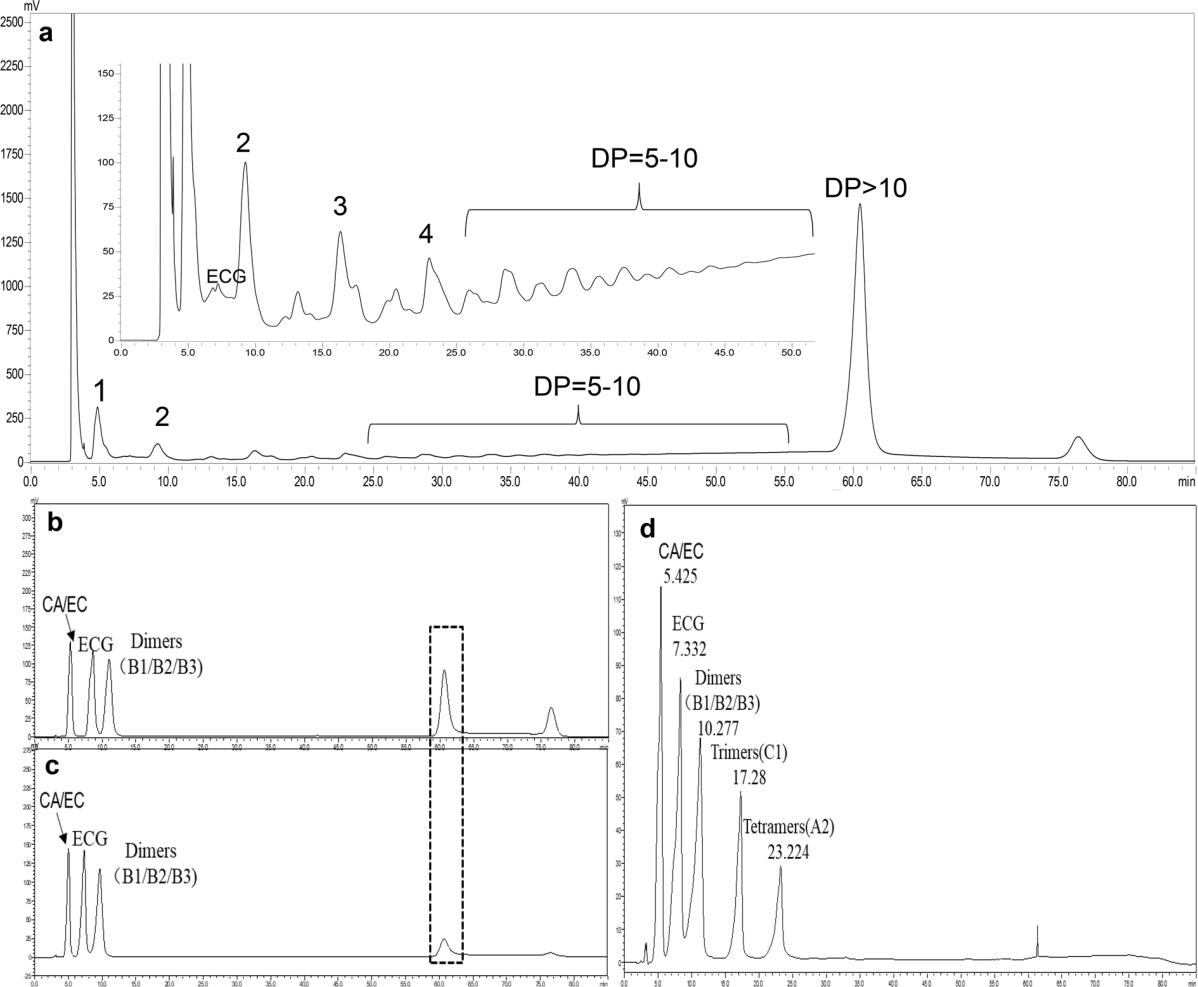
NP-HPLC chromatogram of unexploded grape seeds extract and mixed standard solutions (1 = monomer, 2 = dimers, 3 = trimer, 4 = tetramer) (**a**); mobile phases A = acetonitrile/acetic acid (98:2, v/v) (**b**); mobile phases A = acetonitrile/water (98:2, v/v) (**c**); mixed standard (CA/EC + ECG + Dimers + C1 + A2) (**d**)

Since only solubilized procyanidins in the aqueous phase, such as OPC, are bio-accessible for the enterocyte surface of the small intestine (Ou and Gu 2014), it is necessary to investigate whether the SE treatment could facilitate the depolymerization of PPC, and it is also important to analyze the OPC components after SE treatments. NP-HPLC was applied for procyanidins analysis. Initially, the mobile phase was performed according to Choy’s reports (2013), and the resultant chromatogram indicated that there existed a peak with a big area at 62.2 min, which is also peak position of procyanidins of DP > 10; thus, the operating conditions significantly influenced the measure of procyanidins of DP > 10 (Fig. 4b, c). Nevertheless, when acetic acid in solvent A was replaced by water, the big peak at 62.2 min was reduced substantially, and the measurement of procyanidins with DP ≤ 9 were not affected (Fig. 4b, c, Additional file 1: Table S1). As a result, the solvent A was modified as acetonitrile/water (98/2, v/v). The assignments of the peaks in grape-seeds’ extract were accomplished by comparing their retention time with standards (CA, EC, ECG and procyanidin B1, B2, B3, C1, Cinnamtannin A2) (Additional file 1: Fig. S1a–g) and mixed standards (Additional file 1: Fig. S1h, i) and referring to published data (Additional file 1: Table S2) (Ito et al. 2013; Hellström and Mattila 2008).

**Fig. 5:**
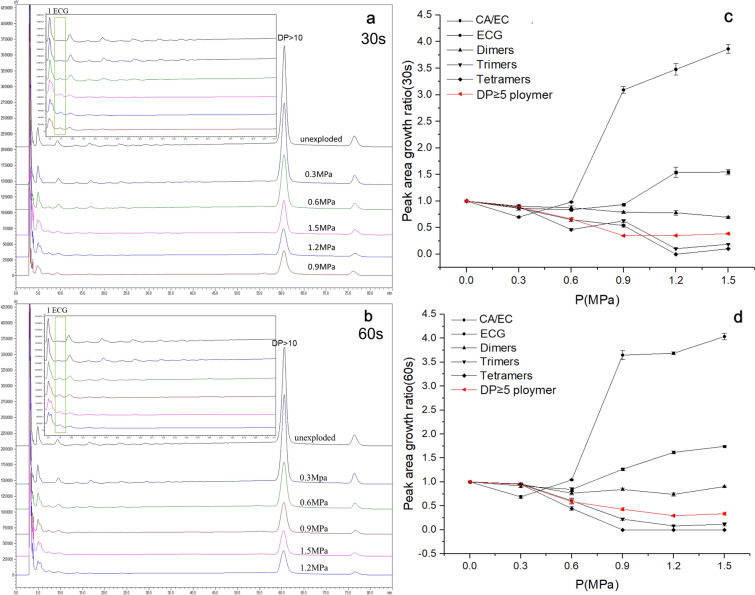
NP-HPLC chromatogram of procyanidins extracted from unexploded and SE-treated grape seeds (**a**, **b**) and the PAGR of unexploded and SE-treated grape seeds (**c**, **d**)

In unexploded grape seeds, procyanidins with DP ≥ 5 accounted for more than 60% of the total procyanidins (data not shown). After SE treatment, most procyanidins with different DP decreased gradually with the increase of SE severity, especially the procyanidins with DP > 10 (Fig. 5a, b), while there is a peak at 7.3 min increasing gradually with the increase of SE severity (Fig. 5a, b, green frame) that was assigned to ECG. Figure 5c, d shows the change of peak area growth ratio (PAGR) of procyanidins with different DP after SE treatments, which was used to describe the effect of SE treatment on procyanidins yield. In Fig. 5c, when the pressure was increased from 0.6 to 1.2 MPa with treating time of 30 s, the PAGR of CA/EC increased from 0.81 to 1.54, and the PAGR of ECG increased from 0.10 to 3.48, indicating a significant increase of CA/EC and ECG. Nevertheless, when the pressure was increased from 1.2 to 1.5 MPa, the increasing trend of the PAGR of CA/EC became slower, while ECG still indicated a higher increasing-rate. The PAGR of procyanidins with DP ≥ 2 all indicated a decreasing trend, especially pressures ranging from 0.6 to 1.2 MPa. The PAGR of dimers showed a slowest decreasing trend in comparison with trimers, tetramers and polymers with DP ≥ 5 (Fig. 5c, d). When treating time was 60 s, the similar results were also observed. These results could be explained by that SE treatment depolymerized the procyanidins to produce monomeric catechins, such as CA, EC and ECG, while lower pressures are not enough to produce them at a large quantity (Fig. 5c, d), which agreed with earlier reports (Gong et al. 2012). It has been reported that SE treatment could degrade the glycoside/ester/ether bonds in plant tissues to release bioactive phytochemicals, and the degrading degree depended on the type of plant tissues and operating parameters of SE treatment (Gong et al. 2012; Chen and Chen 2011). As a result, it could be speculated that the monomeric catechins (CA, EC and ECG) were derived from the depolymerization of procyanidins. On the other hand, SE treatment could also degrade procyanidin to form other compounds that were not detected in the present paper.

The inter-flavanol C–C bonds in procyanidins are easier to be cleaved by strong acids at room temperature or weak acids at higher temperature (Gu et al. 2004). It has been proved wildly that various organic acids were produced after SE treatment, such as levulinic acid, formic acid, acetic acid, etc. (Gong et al. 2012). Figure 5c, d shows that with increase of pressure, the amount of CA/EC and ECG increased and procyanidins with DP ≥ 2 decreased. On the basis of NP-HPLC results, we inferred that during SE treatment the derived organic acids or hydrogen ions would attack the inter-flavanol C–C bonds of PPC at high temperatures (Wojtasz-Mucha et al. 2017), and then CA/EC and ECG were produced from one or both ends of PPC, the inter-flavanol C–C bonds of released intermediate procyanidins would be cleaved further resulting in the production of more CA/EC and ECG; additionally, the resultant CA/EC and ECG might be degraded further (Additional file 1: Fig. S2). Given the abundant source of PPC in plants, it is possible to produce a large number of catechin monomers from PPC with SE treatment. Therefore, SE treatment is a potential effective method to prepare procyanidins with low degree of polymerization, and high antioxidant activity; however, it is notable that SE treatment could also decrease the yield of total procyanidins in the comparison with the unexploded ones (Fig. 1b, c). Consequently, it still needs to study further how to balance of the yield of total procyanidins and oligomeric procyanidins.

## Conclusion

In this work, it was studied how SE treatment influenced the extraction, depolymerization and antioxidant activities of procyanidins from grape seeds. The results indicated that SE caused the loss of procyanidins and decreased TPC of grape-seeds’ extract and mDP of procyanidins. Nevertheless, antioxidant activity of procyanidins and the yield of CA/EC and ECG increased significantly. Consequently, although SE treatment could depolymerize PPC into catechin monomers with high yield, it caused the loss of total procyanidins. SE treatment is a potential effective method to prepared procyanidins with low degree of polymerization and high antioxidant activity. However, it still needs to study further how to balance of the yield of total procyanidins and catechin monomers (CA/EC/ECG).

### Supplementary Information

**Additional file 1: Figure S1**. NP-HPLC chromatogram of procyanidin standards (**a-g**) and mixed procyanidin standards (**h,** **i**). **Figure S2**. Proposed mechanism of PPC depolymerization. **Table S1** Retaining time of procyanidins with different DP in different mobile phase systems. **Table S2**. The identification and peak assignment of procyanidins units after thiolysis.

## Data Availability

They are included within the article and its Additional files.

## Abbreviations

ABTS: 2–20-Azino-di-(3-ethylbenzthiazoline sulfonic acid)
CA: Catechin
CE: Catechin equivalents
DM: Dry matter
DP: Degree of polymerization
DPPH: 2,2-Diphenyl-1-picrylhydrazyl hydrate
EC: Epicatechin
ECG: Epicatechin 3-O-gallate
GA: Gallic acid
GAE: Gallic acid equivalents
mDP: Mean degree of polymerization
OPC: Oligomeric procyanidins
PAGR: Peak area of growth ratio
PPC: Polymeric procyanidins
PC: Procyanidin content
SE: Steam explosion
SEM: Scanning electron microscopy
TE: Trolox equivalent
TPC: Total phenolic content
Trolox: 6-Hydroxy-2,5,7,8-tetramethylchroman-2-carboxylic acid
