# Supplementary material for: Production of oligomeric procyanidins by mild steam explosion treatment of grape seeds
Source: Bioresour Bioprocess. 2021 Mar 23;8(1):23. doi: 10.1186/s40643-021-00376-4 (PMC10992546; doi:10.1186/s40643-021-00376-4)
Supplement: Supplementary file 1 — Additional file 1: Figure S1. NP-HPLC chromatogram of procyanidin standards (a-g) and mixed procyanidin standards (h, i). Figure S2. Proposed mechanism of PPC depolymerization. Table S1 Retaining time of procyanidins with different DP in different mobile phase systems. Table S2. The identification and peak assignment of procyanidins units after thiolysis. [file 40643_2021_376_MOESM1_ESM.docx]

Supporting Information

**Production of oligomeric procyanidins by mild steam explosion treatment of grape seeds**

Jie Zhang^1, 2, #^, Dan Liu^1, #^, Aoke Wang^1^, Li Cheng^1^, Wenya Wang^1, 2, *^, Yanhui Liu^1^, Sadeeq Ullah^1^, and Qipeng Yuan^1,*^

*Correspondence: [wangwy@mail.buct.edu.cn](mailto:wangwy@mail.buct.edu.cn); [yuanqp@mail.buct.edu.cn](mailto:yuanqp@mail.buct.edu.cn)

^1^College of Life Science and Technology, Beijing University of Chemical Technology, Beijing 100029, China

^2^Amoy-BUCT Industrial Bio-Technovation Institute, Amoy 361022, China

^#^These authors contribute to the work equally.


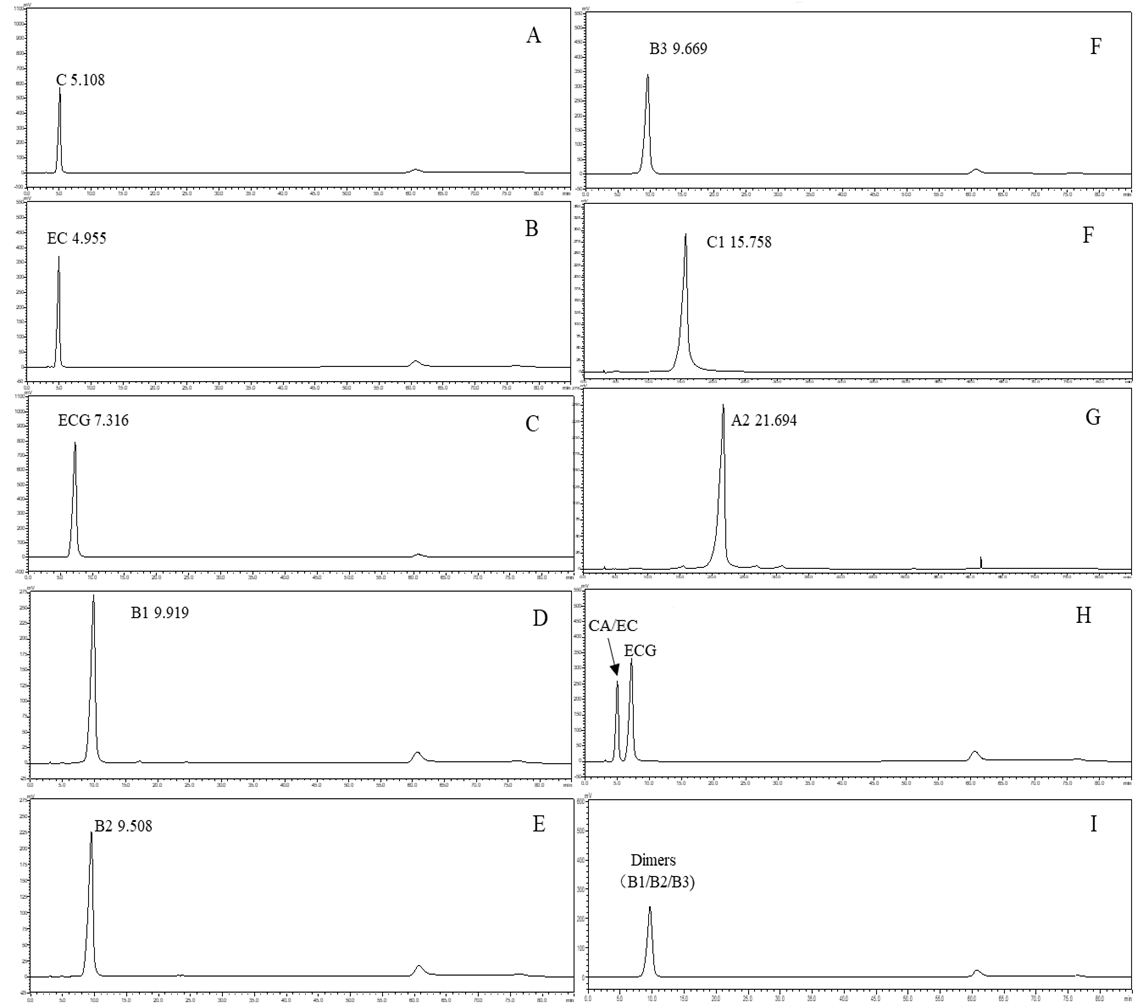


**Fig. S1** NP-HPLC chromatogram of procyanidin standards (A-G) and mixed procyanidinstandards (H, I).

**
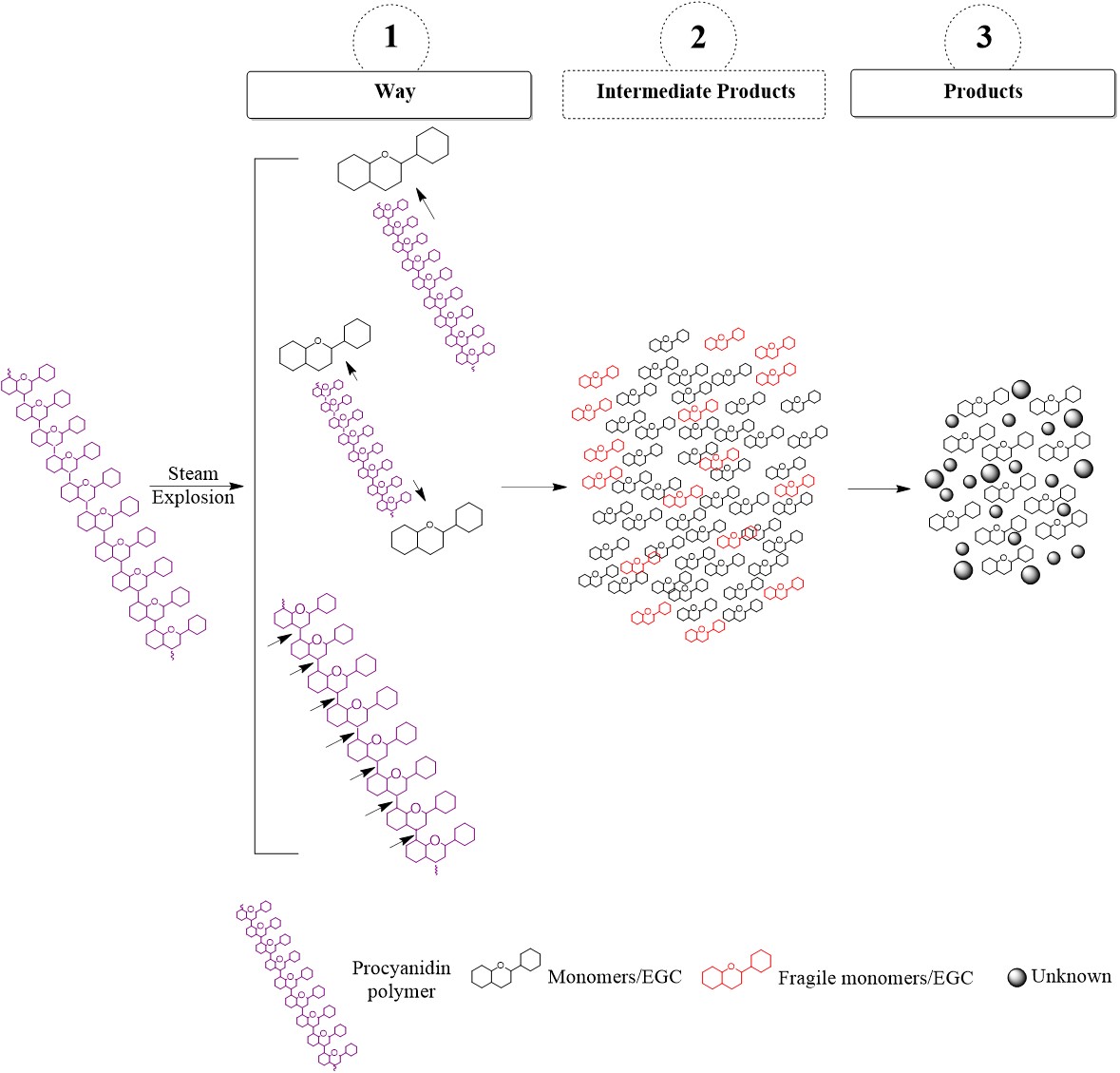
**

**Fig. S2** Proposed mechanism of PPC depolymerization.

**Table S1** Retaining time of procyanidins with different DP in different mobile phase systems.

| DP | Residence time (min) - acetonitrile：acetic acid =98:2 (Choy et al. 2013) | Residence time (min) – acetonitrile：water =98:2 |
| --- | --- | --- |
| 1 | 4.9 | 5.4 |
| ECG | - | 7.3 |
| 2 | 10.2 | 10.3 |
| 3 | 17.3 | 17.3 |
| 4 | 23.9 | 23.2 |
| 5 | 28.9 | - |
| 6 | 33.4 | - |
| 7 | 37.5 | - |
| 8 | 41.5 | - |
| 9 | 44.5 | - |
| 10 | 46.5 | - |
| >10 | 56.7 | - |

**References**

Choy YY, Jaggers GK, Oteiza PI, Waterhouse AL (2013) Bioavailability of intact proanthocyanidins in the rat colon after ingestion of grape seed extract. J Agr Food Chem 61(1):121-127

**Table S2** The identification and peak assignment of procyanidins units after thiolysis

| Peak | RT^*^ (min) | Compound | Identification method |
| --- | --- | --- | --- |
| 1 | 10.3 | catechin | Standard, Figure 2B |
| 2 | 14.8 | epicatechin | Standard, Figure 2C |
| 3 | 18.1 | epicatechingallate | Standard, Figure 2D |
| 4 | 28.4 | 3,4-trans-catechin benzylthioether | Standard, Figure 2G  (Hellström et al. 2008; Sun et al. 2011) |
| 5 | 29.1 | (epi) gallocatechin benzylthioether | (Hellström et al. 2008; Çam et al. 2010; Vivas et al. 2004) |
| 6 | 30.4 | 3,4-cis-catechin benzylthioether | Standard, Figure 2G  (Hellström et al. 2008; Sun et al. 2011) |
| 7 | 33.7 | 3,4-trans-epicatechin benzylthioether | Standard, Figure 2F  (Hellström et al. 2008; Çam et al. 2010) |
| 8 | 35.2 | epicatechin 3-O-gallate benzylthioether | (Hellström et al. 2008; Çam et al. 2010; Vivas et al. 2004; Kruger et al. 2014) |
| 9 | 37.4 | benzylmercaptan | Standard, Figure 2E |

Note: RT^*^ means retention time

**References**

Çam M, Hışıl Y (2010) Pressurised water extraction of polyphenols from pomegranate peels. Food Chem123(3):878-885

Hellström JK, Mattila PH (2008) HPLC determination of extractable and unextractable proanthocyanidins in plant materials. J Agr Food Chem 56(17):7617-7624

Kruger MJ, Davies N, Myburgh KH, Lecour S (2014) Proanthocyanidins, anthocyanins and cardiovascular diseases. Food Res Int 59:41-52

Sun B, Neves AC, Fernandes TA, Fernandes AL, Mateus N, De Freitas V, Leandro C, Spranger MI (2011) Evolution of phenolic composition of red wine during vinification and storage and its contribution to wine sensory properties and antioxidant activity. J Agr Food Chem 59(12):6550-6557

Vivas N, Nonier MF, De Gaulejac NV, Absalon C, Bertrand A, Mirabel M (2004) Differentiation of proanthocyanidin tannins from seeds, skins and stems of grapes (*Vitis vinifera*) and heartwood of Quebracho (*Schinopsis balansae*) by matrix-assisted laser desorption/ionization time-of-flight mass spectrometry and thioacidolysis/liquid chromatography/electrospray ionization mass spectrometry. Anal Chim Acta 513(1):247-256
